# Gastric bezoar: a simple and innovative lithotripsy method—a case report and review of the literature

**DOI:** 10.3389/fgstr.2025.1527031

**Published:** 2025-04-01

**Authors:** Xudong Yan

**Affiliations:** Department of Gastroenterology, Affiliated Hospital and Clinical Medical College of Chengdu University, Chengdu, Sichuan, China

**Keywords:** bezoar, lithotripsy, innovation, zebra guidewire, review

## Abstract

Gastrointestinal bezoars result from the ingestion of indigestible materials and are classified by their consistency. This material is commonly located in the stomach. Human bezoars may be formed by non-digestible plant fibers (phytobezoars), persimmons (diospyrobezoar), hair (trichobezoar), long-acting medications (pharmacobezoar), milk and mucus components (lactobezoar), or other various substances. This condition may be asymptomatic, or it may cause symptoms such as stomachache, ulcers, bleeding, perforation, gastric outlet obstruction, and mechanical intestinal obstruction. We herein report a simple and innovative lithotripsy method using Zebra Guidewire, and review the pathogenesis, clinical manifestations, and treatment of bezoars.

## Introduction

Gastric bezoars are defined as foreign bodies accumulating in the stomach (1). Gastric bezoars cause non-specific symptoms and are often incidentally discovered in patients undergoing upper gastrointestinal endoscopy or computed tomography (CT) examination. This article will report a simple and innovative lithotripsy method using Zebra Guidewire and review related literature.

## Case presentation

**Figure 1:**
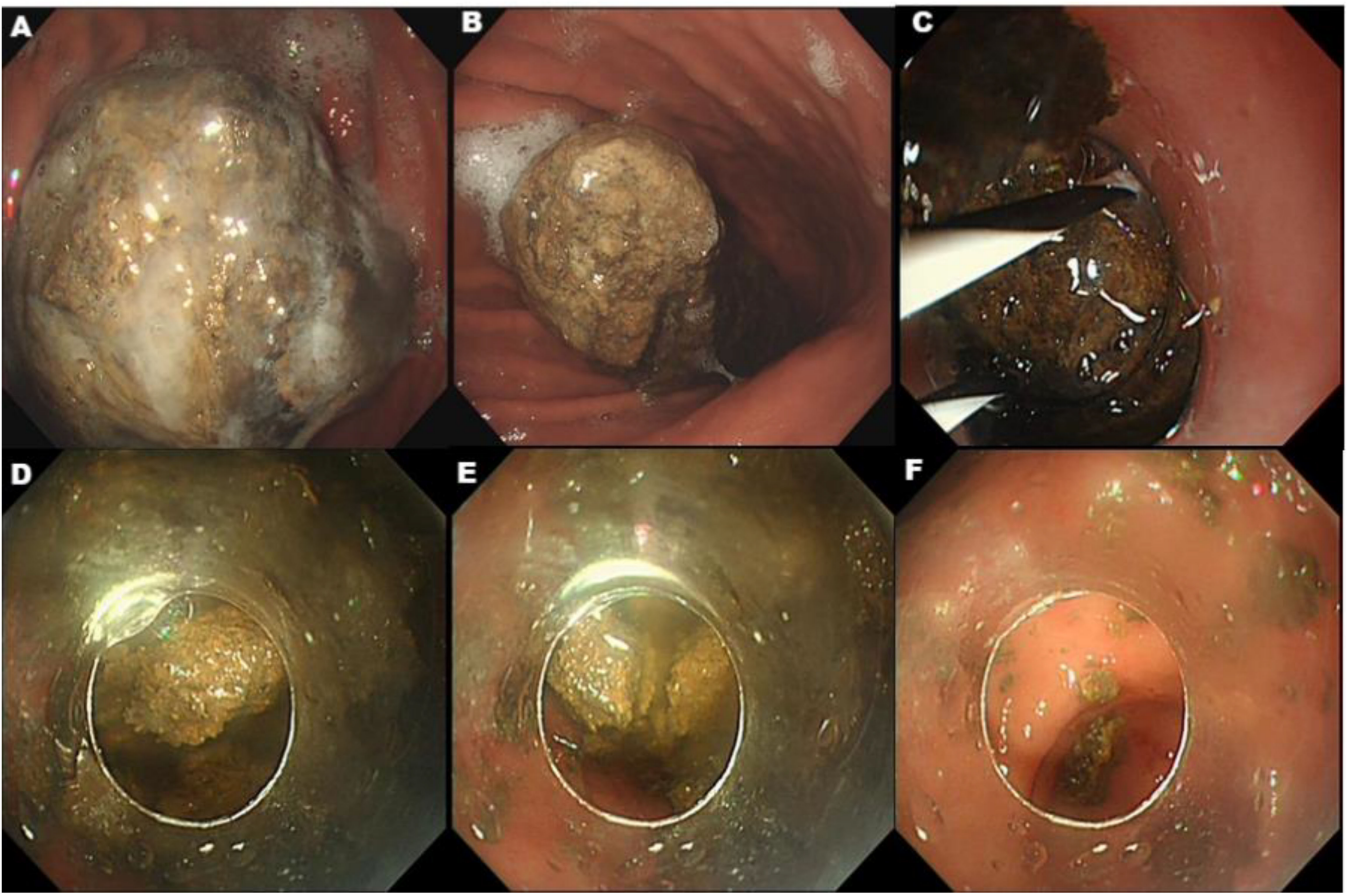
**(A, B)** A gastrolith surface adhesive mucus measuring about 5 cm × 7 cm was visible in the body of the stomach, which was dark brown in color and hard in texture. **(C)** After the Zebra Guidewire covered the gastric bezoar, we retracted both ends of the guidewire for mechanical fragmentation of the bezoar. **(D, E)** Repeated lithotripsy of gastric bezoar into many small fragments that were able to pass through the pylorus. **(F)** All of the gastric bezoar was removed.

**Figure 2:**
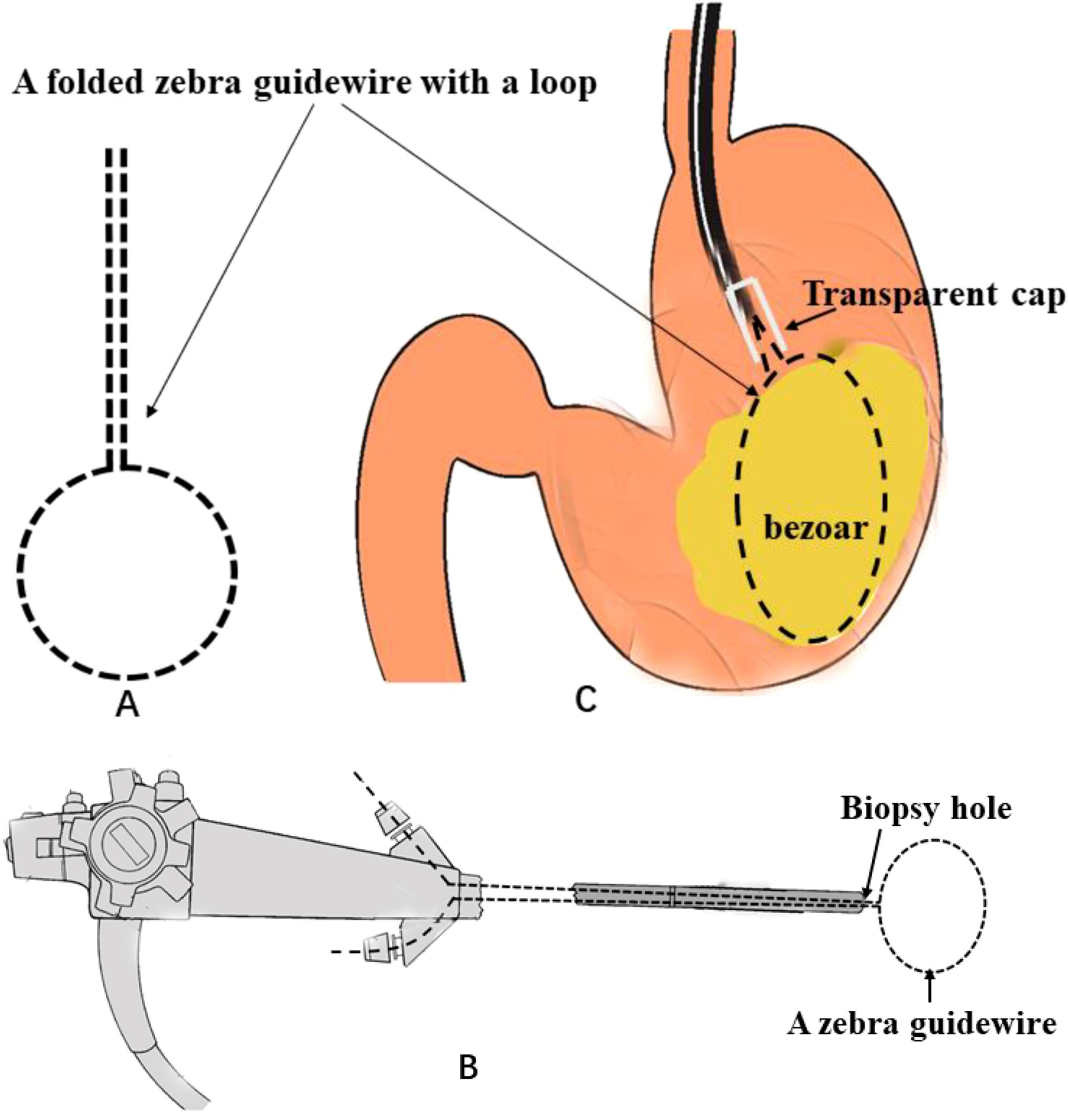
Schematic diagram of how a giant gastric bezoar was removed using a folded Zebra Guidewire. **(A)** A folded Zebra Guidewire. **(B)** The folded Zebra Guidewire was inserted into a double-channel gastroscopy. **(C)** The process of removal bezoar.

A 69-year-old woman was referred to our hospital with epigastric pain for 20 days. The patient reported a long habit of eating persimmons. Her physical examination and laboratory investigations were normal except for upper abdominal tenderness. Gastroscopy revealed a giant hard bezoar in the stomach, with an approximate size of 5.0 cm × 7.0 cm and a smooth surface, which was impossible to remove directly using standard retrieval devices (**Figures 1 A, B**). On the second day after admission, a long transparent cap was first placed at the front end of the Olympus 260 double-channel gastroscope. A folded Zebra Guidewire with a loop (**Figure 2A**) was inserted into the gastroscope along the direction of the biopsy channel, and then the gastroscope with the Zebra Guidewire was inserted into the gastric cavity. Since the size of the snare loop can be adjusted according to the volume of the bezoar (**Figure 2B**), the massive bezoar was successfully captured and secured by the guidewire under endoscopic guidance (**Figure 2C**). Initially, the bezoar was pulled towards the transparent cap, followed by simultaneously tightening both ends of the guidewire with force. This mechanical force effectively cut the bezoar into two pieces (**Figure 1C**). The above steps were repeated multiple times until the maximum diameter of the gastroliths was less than 2.0 centimeters (**Figures 1D, E**) and could pass through the pylorus freely. The bezoar was successfully removed without damaging the gastric mucosa (**Figure 1F**). Subsequently, the patient’s epigastric pain was relieved and no complications occurred. The entire procedure took nearly 2 hours under anesthesia. A week later, endoscopic examination revealed multiple gastric ulcers, but no remnants of the bezoar were found. The whole process is shown in **Figures 1**, **2** provides a schematic diagram.

### Pathogenesis

Bezoars can be categorized as phytobezoars, trichobezoars, pharmacobezoars, and others, according to their composition. Phytobezoars, mainly composed of indigestible plant fibers, are the most common type of bezoars. At present, there are two kinds of hypotheses about the cause of phytobezoars. First, unripe persimmons contain a large amount of tannic acid. Tannic acid molecules combine with gastric mucin in an acidic environment to form insoluble tannic acid proteins. These tannic acid proteins further combine with pectin, cellulose, and other high-fiber substances (e.g., citrus fruits, peels, and seeds) to form polymers. These polymers continually combine with each other to form macromolecular polymers, thereby leading to the formation of bezoars (2). Second, persimmon tannins themselves are prone to self-aggregation and can easily combine with proteins to form precipitates (3). Compared to insoluble persimmon tannins, soluble persimmon tannins are more likely to form complexes with food that have a higher turbidity, thus making flocculation more likely to occur (4). In addition, when the precipitates were added to simulated gastric fluid (SGF), Coca-Cola, and 5% sodium bicarbonate solution, respectively, the results showed that all the precipitates formed by soluble persimmon tannins were completely dissolved by the SGF, but neither Coca-Cola nor the 5% sodium bicarbonate solution could dissolve them. Therefore, the formation of precipitates does not mean there is a risk of forming bezoars. It is believed that healthy adults who consume 400–500 grams of persimmons each day will not develop gastric bezoars (4).

In people with normal gastric emptying, the problem may arise from abnormal grinding of the stomach, and gastric bezoars may be a temporary problem in many cases (5). In addition to the dietary factors mentioned above, the mechanism of bezoar formation is also related to primary and secondary digestive tract anatomical abnormalities, abnormal gastric motility, or other systemic complications.

### Clinical manifestations

Gastric bezoars are usually discovered accidentally when patients undergo imaging or endoscopic examination for non-specific symptoms. On an abdominal CT scan, gastric bezoars have the appearance of a low-density intraluminal mass containing air bubbles and exhibit a characteristic mottled appearance (6). Endoscopy is considered the most accurate diagnostic modality in suspected patients.

The affected patients are either asymptomatic or exhibit various gastrointestinal symptoms. The most common symptoms include abdominal pain, nausea, vomiting, early satiety, anorexia, and weight loss (7). The incidence of ulcers in patients with gastric bezoar is 52.9% (8). Compared with peptic ulcers, mechanical ulcers caused by gastric bezoars are more likely to lead to gastrointestinal bleeding. Moreover, the ulcer healing rate caused by gastric bezoar is relatively high.

### Management

The treatment of gastric bezoar mainly includes endoscopic treatment, drug therapy, and surgical treatment. The instruments commonly used in endoscopic treatment include foreign-body forceps, rat-bite forceps, alligator forceps, net baskets, and snares. Gastric bezoars can be removed directly with a net basket or a snare. Larger stones that cannot pass through the pylorus can be removed by mechanical lithotripsy. Rat-bite pliers and alligator-bite pliers are used to crush the surface and sodium bicarbonate is injected into different parts. It is then crushed by methods such as gravel net basket and snare cutting. The larger stones are removed and the small stones can be discharged by themselves (9).

The choice of endoscopic or surgical lithotomy depends on the size and composition of the bezoar. There are two main difficulties that arise in endoscopic lithotomy. First, when the volume of gastric bezoar is very large, it cannot pass through the narrow esophagus. The second is that the main components of bezoars are food fibers or human hair, which are very tough and dense and cannot be separated by simple mechanical cutting methods. Therefore, auxiliary high-frequency electrical cutting can be used. Hair itself cannot withstand high temperatures, so after the action of electrocoagulation, a snare is used, coupled with external cutting, and the gastric hairball is gradually cut smaller and separated. However, the biggest complication of this method is the damage to the gastric mucosa caused by electrocoagulation during the cutting process, which can even lead to perforation in severe cases. Therefore, during the operation, the operation time should be shortened as much as possible to avoid contact between the cutting site and the stomach wall, and combined with gastric mucosal protective agents and proton pump inhibitors (PPI) to promote the repair of the mucosa after surgery.

Compared with the snare and gravel net basket, the Zebra Guidewire not only has a lower cost but also greatly reduces the cost of surgical consumables and patient hospitalization expenses. The advantages are as follows. ① The zebra guidewire is adjustable and has good flexibility. Ordinary instruments such as a snare may be deformed after multiple attempts when dealing with large gastric stones, while the Zebra Guidewire is not easily deformed during repeated gravel process and can be reused. Regardless of the diameter of the bezoar, the Zebra Guidewire can be used to repeatedly crush it, which greatly improves the success rate of lithotripsy. It is especially suitable for large phytobezoars ≥4cm, which cannot be effectively wrapped by snares or lithotripsy baskets. ② For bezoars that have been formed over a long duration and are hard in texture, the snare or basket will deform after being used 1-2 times, and it cannot be fully opened again and thus loses its function, so a new snare or basket must be used. The Zebra Guidewire can be used by changing the gravel part until the gravel part of the guidewire is completely deformed.

Chemical dissolution plays an important role in the treatment of gastric bezoar. The advantage of chemical dissolution is that it is non-invasive and economical. Administering Coca-Cola (3,000 mL within 12 hours) through a nasogastric tube or by drinking has been used for chemical dissolution (10). The mechanism of how Coca-Cola beverages dissolve bezoars is not fully understood, but the suspected mechanisms include 1) the mucolytic effect of its high sodium bicarbonate content; 2) acidification by carbonic acid and phosphoric acid; 3) destruction of bezoar structure by carbon dioxide bubbles (11–13). Oral administration of 5% sodium bicarbonate can release carbon dioxide under the action of stomach acid, promoting the loosening and decomposition of stomach bezoars (14). Even in patients who have been treated with endoscopic lithotripsy, bezoars are still likely to reaggregate into clusters, and chemical-assisted treatment is also required. If residual stones are found in the gastroscopy examination after 2 weeks of conservative treatment, it indicates that the conservative drug treatment is ineffective and endoscopic lithotripsy should be added. However, the consistency of the gastric bezoars is usually very hard at this time, which increases the difficulty of lithotripsy.

Surgical treatment can directly remove gastric bezoars (15), but due to its associated trauma, high cost, long hospital stay, and high risk of postoperative complications, it is more suitable for patients with trichobezoars or very large bezoars that cause obstruction and cannot be removed with a Roth net or strong suction.

## Conclusion

The instruments used in the Zebra Guidewire with a transparent cap lithotripsy method are simple and easy to obtain, and the operation process is simple and easy to master. The lithotripsy duration is shorter, the efficiency is higher, and the trauma is smaller. Using a transparent cap at the front end of the gastroscope can create a buffer distance between the Zebra Guidewire and the lens, thereby avoiding damage to the tip of the gastroscope during the process of cutting the bezoars. This method may become a suitable option for the endoscopic removal of gastric bezoars.

## Data Availability

The datasets presented in this study can be found in online repositories. The names of the repository/repositories and accession number(s) can be found in the article/supplementary material.
